# Automated Classification of Enamel Caries from Intraoral Images Using Deep Learning Models: A Diagnostic Study

**DOI:** 10.3390/jcm14248959

**Published:** 2025-12-18

**Authors:** Faris Yahya I. Asiri

**Affiliations:** Department of Preventive Dental Sciences, College of Dentistry, King Faisal University, Al Ahsa 31982, Saudi Arabia; fasiri@kfu.edu.sa

**Keywords:** dental caries, artificial intelligence, machine learning, diagnostic imaging, preventive dentistry, dental public health

## Abstract

**Background:** Dental caries is a prevalent global oral health issue. The early detection of enamel caries, the initial stage of decay, is critical to preventive dentistry but is often limited by the subjectivity and variability of conventional diagnostic methods. **Objective:** This study aims to develop and evaluate two explainable deep learning models for the automated classification of enamel caries from intraoral images. **Dataset and Methodology**: A publicly available dataset of 2000 intraoral images showing early-stage enamel caries, advanced enamel caries, no-caries was used. The dataset was split into training, validation, and test sets in a 70:15:15 ratio, and data preprocessing and augmentation were applied to the training set to balance the dataset and prevent model overfitting. Two models were developed, ExplainableDentalNet, a custom lightweight CNN, and Interpretable ResNet50-SE, a fine-tuned ResNet50 model with Squeeze-and-Excitation blocks, and both were integrated with Gradient-Weighted Class Activation Mapping (Grad-CAM) for visual interpretability. **Results**: As evaluated on the test set, ExplainableDentalNet achieved an overall accuracy of 96.66% and a Matthews Correlation Coefficient [MCC] = 0.95, while Interpretable ResNet50-SE achieved 98.30% accuracy (MCC = 0.975). McNemar’s test indicated no significant prediction bias, with *p* > 0.05, and internal bootstrap and cross-validation analyses indicated stable performance. **Conclusions**: The proposed explainable models demonstrated high diagnostic accuracy in enamel caries classification on the studied dataset. While the present findings are promising, future clinical applications will require external validation on multi-center datasets.

## 1. Introduction

Enamel caries, the early demineralization and breakdown of the tooth’s outer enamel layer, represents a fundamental stage in the caries disease continuum, preceding cavitation and more extensive dentine involvement [1]. Dental caries is the most common non-communicable disease globally, with the untreated decay of permanent teeth being the single most prevalent health condition, according to the World Health Organization (WHO) [2]. Epidemiological studies indicate that enamel-level caries constitutes approximately 50% of all proximal lesions in adolescents aged 12 to 15 years [3,4]. In England, a substantial proportion of 5-year-old children had evidence of enamel and dentinal decay [5]. From a public health perspective, the early detection of enamel caries is crucial because timely preventive interventions can reduce progression, preserve tooth structure, and reduce the burden on dental healthcare systems [6].

Traditionally, the diagnosis of enamel caries relies on visual, tactile inspection, radiographic images, and adjunctive technologies such as fluorescence and transillumination [7]. However, these methods have limitations, including visual examination may miss subsurface lesions, radiographs expose patients to ionizing radiation, and interpretation often heavily depends on clinician expertise, resulting in variability and potential under- or overdiagnosis [8,9]. Furthermore, in large-scale dental public health screening, the manual diagnostic process is labor-intensive and subject to examiner fatigue, which limits scalability and consistency in outcomes.

Against this backdrop, the need for automated, accurate, and interpretable diagnostic systems for enamel caries has emerged. Recently, Artificial Intelligence (AI) has emerged as a useful tool for dental diagnosis [10]. Deep learning models can analyze radiographs and clinical images to identify subtle features that may be invisible to the human eye [11,12,13,14]. Generative Adversarial Networks (GANs) have also been employed to synthesize realistic dental images, thereby augmenting limited datasets and improving the training of deep learning models [15]. Emerging AI tools, particularly those based on deep learning, offer transformative potential: the image-based detection of enamel lesions, personalized caries risk prediction, and even virtual training to enhance clinical decision making [16,17,18]. Advances in Artificial Intelligence (AI) offer transformative opportunities in dental diagnostics [19,20]. Convolutional neural networks (CNNs) have shown promising results in image-based caries detection [21]. Kühnisch et al. [22] utilized 2417 intraoral images to develop a CNN for caries detection, achieving 92.5% accuracy and an AUC of 96.4. However, further improvement is needed, as such high performance was primarily achieved on a standardized single-tooth image.

Moreover, Park et al. [23] developed a U-Net and Faster R-CNN model and trained it on intraoral camera images for tooth surface segmentation, with training, validation, and test set of 1638, 410, and 300 images, respectively; the results showed improved caries classification, with 83.37% AUC. Similarly, Lee et al. [21] trained a CNN-based U-Net model on 304 bitewing radiographs; the achieved recall of 65.02% showed an improvement of the model over clinicians’ sensitivity, but 63.29% precision indicated a high rate of false positives. In a previous study, Kang et al. [24] evaluated the ensemble CNN-based methods ResNet-50, Inception-v3, InceptionResNetv2, and Faster-R-CNN on 2682 intraoral camera images, achieving maximum AUC of 94% and average precision of 97%. Similarly, ForouzeshFar et al. [25] used 713 patient bitewing images and reported that the VGG19 model achieved the highest accuracy of 93.93% in caries diagnosis. Furthermore, Esmaeilyfard et al. [26] utilized a CBCT dataset of 785 molar teeth to develop a multiple-input CNN model which achieved a diagnostic accuracy of 95.3% in caries detection. However, few studies have focused on classifying early- and advanced-stage enamel caries in standard intraoral photographs with models that prioritize both high accuracy and clinical interpretability based on explainable AI techniques, a necessary combination for building trust and facilitating adoption in public health screening. Furthermore, while RGB intraoral images are widely available and cost-effective, emerging multimodal imaging approaches such as Quantitative Light-induced Fluorescence (QLF), near-infrared transillumination, and laser fluorescence offer enhanced sensitivity in detecting early demineralization and dental plaque, which are precursors to caries. Integrating such multimodal data could improve early caries risk assessment and personalize preventive interventions. However, the development of explainable AI models for multimodal caries detection remains underexplored. Therefore, a significant gap exists in the development of highly accurate, explainable deep learning systems specifically designed for early enamel caries detection from intraoral imagery that are both statistically reliable and clinically transparent for public health screening. This study aimed to (i) develop and evaluate two deep learning architectures, ExplainableDentalNet and Interpretable ResNet50-SE, for the automatic classification of enamel caries stages; (ii) integrate Grad-CAM to provide visual explanations for model predictions; and (iii) statistically compare the models’ diagnostic performance and stability, based on the hypothesis that Interpretable ResNet50-SE would achieve superior accuracy due to its advanced attention mechanism. Our major contributions are as follows:

1. We propose a lightweight CNN-based model, ExplainableDentalNet, which incorporates multi-scale feature extraction and Grad-CAM interpretability to improve diagnostic transparency in enamel caries detection.
2. We propose Interpretable ResNet50-SE, a model based on a fine-tuned ResNet50 architecture with Squeeze-and-Excitation blocks and a 1024-D deep feature layer to enhance classification accuracy and feature interpretability.
3. We implement comprehensive statistical validation, such as McNemar’s tests and bootstrapped stability analyses, to assess class-wise reliability and diagnostic performance.

By bridging dental public health needs with advanced AI methodologies, this work contributes a scalable, interpretable diagnostic AI-based framework for enamel caries detection and highlights its potential role in preventive dentistry.

## 2. Materials and Methods

**Figure 1:**
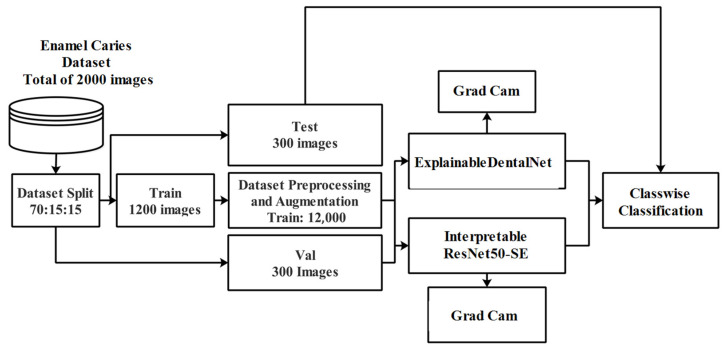
Proposed workflow of the explainable deep learning framework for enamel caries classification, integrating data preprocessing, augmentation, and the ExplainableDentalNet and Interpretable ResNet50-SE models for automated diagnosis.

The workflow of the present study involves several key stages, as shown in Figure 1. Initially, publicly available dental images were collected and organized into relevant classes, and the dataset was split into training (70%), validation (15%), and test (15%) sets. Then, data preprocessing and augmentation were performed to enhance image quality and ensure consistency, followed by techniques such as rotation, flipping, and scaling to improve model generalization on the training set. Two deep learning models were developed for enamel caries classification and explainability: ExplainableDentalNet and Interpretable ResNet50-SE. Explainable AI techniques, such as Grad-CAM visualization, were integrated to enable the interpretability of the model predictions based on the clear visualization of regions influencing diagnostic decisions. This study followed the Checklist for Artificial Intelligence in Medical Imaging (CLAIM)-2024 [27,28] and STARD-2015 guidelines [29] to ensure methodological rigor and reproducibility (see Supplementary Materials).

### 2.1. Dataset Collection

A publicly available dataset was utilized to develop and evaluate the proposed framework [30]. It consists of 2000 low-resolution 224 × 224 × 3 intraoral images in JPG format, categorized into three diagnostic classes: Advanced Enamel Caries, with 800 images; Early-Stage Enamel Caries, with 800 images; and No Enamel Caries, with 400 images. The dataset was collected by American International University, Bangladesh, and the Bangladesh University of Business and Technology and has been made available under a Creative Commons Attribution 4.0 International (CC BY 4.0) license [30]. As the dataset is anonymized and publicly accessible, ethical approval was not required for its use in this research study. The diagnostic labels for the original dataset were provided by its authors [30]. However, the associated publication does not specify the detailed annotation protocol, including the number and experience level of the dental annotators, the specific diagnostic criteria (e.g., whether the ICDAS system was used), or the process for reaching a consensus on disputed cases. This lack of granular metadata regarding ground-truth establishment is a recognized limitation of the source data. To verify label consistency for our study, a random sample representing 10% of the dataset (*n* = 200 images) was independently reviewed by an experienced dental clinician. Inter-rater agreement between the original dataset labels and this independent review yielded a Cohen’s Kappa (κ) of 0.85, indicating substantial agreement and supporting the usability of the labels for this proof-of-concept study.

### 2.2. Dataset Splitting

The complete dataset was divided into three subsets, training, validation, and test sets, in a 70:15:15 ratio using a stratified random split to preserve class balance. To prevent data leakage, the split was performed before data preprocessing and augmentation, ensuring that no image or its augmented variant appeared in more than one subset.

#### 2.2.1. Data Preprocessing and Augmentation

**Figure 2:**
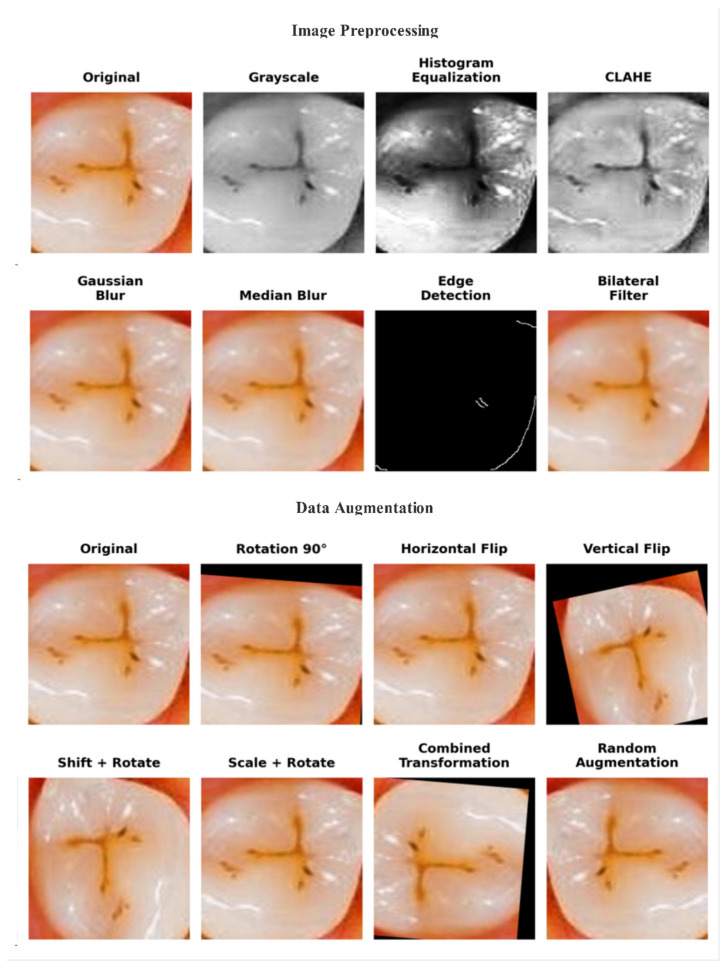
Visual comparison of original image and preprocessed and augmented images.

Several preprocessing and augmentation procedures were applied to enhance data quality and model generalization. First, all images were resized to 224 × 224 pixels to ensure uniform input image dimensions. Then, the preprocessing steps of histogram equalization, CLAHE, Gaussian and median blurring, morphological filters, and normalization were performed to improve image contrast, reduce noise, and emphasize the structural details of enamel surfaces, as shown in Figure 2.

After image preprocessing, data augmentation was applied to the training set to enhance model generalization and minimize overfitting. The augmentation pipeline included random rotations of ±20°, horizontal and vertical flips, scaling within a range of 0.9 to 1.1×, and shifts of ±10% in image width and height. These parameters were chosen to reflect realistic clinical variations while preserving diagnostic features, leading to diverse diagnostic variations while maintaining enamel integrity [31]. The validation and test sets remained unaltered to ensure unbiased evaluation. Following augmentation, the training set increased from 1400 to 12,000 images; this step was implemented to balance the classes, with the ‘No Enamel Caries’ class increasing from 200 to 4000 samples. The final dataset distribution is summarized in Table 1.

#### 2.2.2. Deep Learning Models

Two deep learning architectures were developed and evaluated for the automated classification of dental caries: a custom lightweight model, ExplainableDentalNet, and an interpretable ResNet50-based model with Squeeze-and-Excitation (SE) attention. Their architectural details are illustrated in Supplementary Figures S1 and S2, respectively, which outline the complete layer-wise design and detection flow.

#### 2.2.3. ExplainableDentalNet Architecture

ExplainableDentalNet is a novel AI model developed to support the clinical detection of enamel caries while remaining transparent and easy to interpret. The system processes standard dental images and extracts visual cues that mirror those used by clinicians, including changes in surface texture, brightness, and early structural irregularities. The model’s multi-scale feature design enables it to evaluate each region from different observational perspectives, helping distinguish early demineralization from more advanced lesions. A stabilizing learning mechanism ensures consistent diagnostic behavior across varied tooth surfaces and image quality levels, and the architecture also enhances sensitivity to fine enamel changes that represent the earliest signs of disease progression. Based on these refined features, the model classifies teeth into early-stage enamel caries, advanced enamel caries, or sound enamel. A major contribution is the model’s built-in explainability, which enables the visualization of the exact regions influencing its predictions, thereby supporting clinical trust, validation, and informed decision making.

#### 2.2.4. Interpretable ResNet50-SE Architecture

The second model was built on a ResNet50 backbone and enhanced with a Squeeze-and-Excitation attention module to improve its ability to highlight clinically relevant features. The pretrained ResNet50 network served as a stable foundation, with early layers being kept fixed to retain general image recognition capabilities, while the later layers were fine-tuned to dental-specific patterns. The SE module strengthens the model’s focus by enabling it to emphasize the image regions that contribute the most to enamel caries recognition. After feature extraction, the system summarizes the information with global pooling operations, producing a consolidated, high-dimensional feature representation. This representation was used both for classification and for assessing model stability with bootstrap and cross-validation procedures. The final classification layers include regularization steps to ensure reliable performance and generate predictions for early-stage enamel caries, advanced enamel caries, or sound enamel.

#### 2.2.5. Hyperparameters

The ExplainableDentalNet model was trained for 100 epochs using the Adam optimizer with a learning rate of 0.001, a batch size of 32, and sparse categorical cross-entropy loss. To prevent overfitting, Early Stopping and learning rate reduction on plateau (ReduceLROnPlateau) were employed with patience values of 10 and 8, respectively.

**Figure 3:**
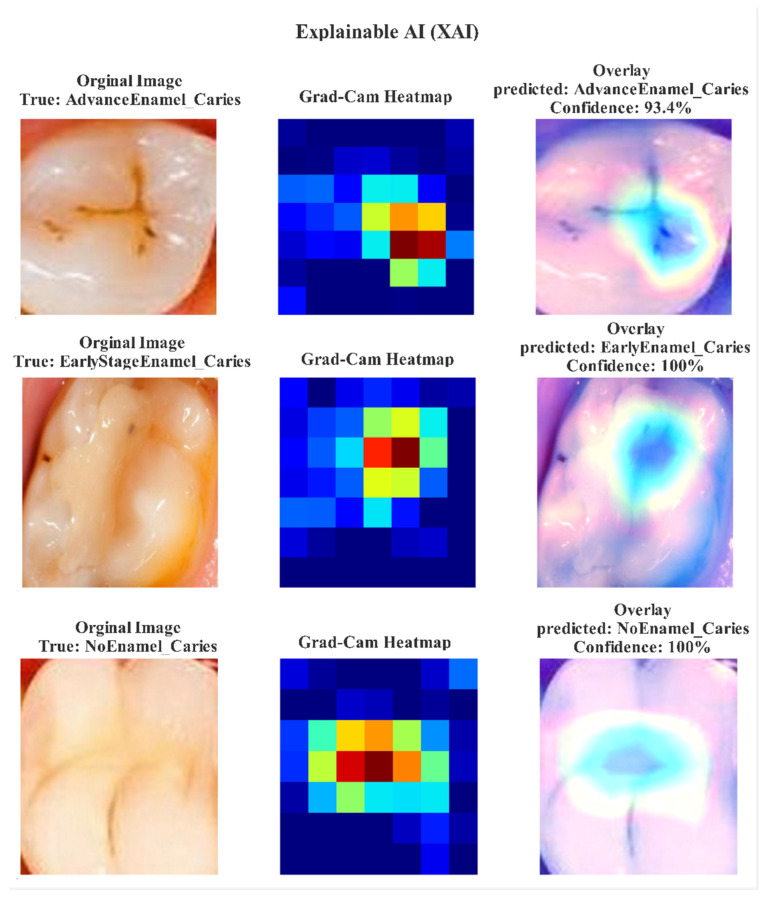
Grad-CAM visualization results demonstrating the attention focus of ExplainableDentalNet and Interpretable ResNet50-SE models on early-stage and advanced enamel caries lesions. Warmer regions indicate that model attention contributed to diagnostic decision making.

For the Interpretable ResNet50-SE model, training was also conducted for 100 epochs with a learning rate of $1\times 10^{-4}$, a batch size of 32, and the same Adam optimizer and loss function. A combination of Model Checkpoint, Early Stopping (patience = 15), and ReduceLROnPlateau callbacks was used to maintain training stability. Both models utilized data augmentation techniques such as rotations, horizontal flips, shifts, and zoom transformations to enhance generalization and reduce dataset bias. All experiments were conducted using TensorFlow (Google LLC, Mountain View, CA, USA) via the Keras API on the Kaggle platform (Kaggle Inc., San Francisco, CA, USA) within a GPU P100 environment. To enhance model interpretability, Gradient-Weighted Class Activation Mapping (Grad-CAM) was employed to visualize the regions that influenced classification decisions for each dental image [32]. These visualizations highlighted the enamel caries regions most responsible for diagnostic lesions, as shown in Figure 3. The Grad-CAM overlays show that both models accurately focused on the relevant caries regions, which supports their diagnostic transparency and reliability [33].

## 3. Results

To assess the performance of both models, ExplainableDentalNet and Interpretable ResNet50-SE, several standard performance metrics were utilized, i.e., precision, recall, F1-score, and accuracy [34], according to the following formulas:

(1) $$\mathrm{Precision}=\frac{TP}{TP+FP}$$

(2) $$\mathrm{Recall}\;(\mathrm{Sensitivity})=\frac{TP}{TP+FN}$$

(3) $$F1\text{-}\mathrm{Score}=2\times \frac{\mathrm{Precision}\times \mathrm{Recall}}{\mathrm{Precision}+\mathrm{Recall}}$$

(4) $$\mathrm{Accuracy}=\frac{TP+TN}{TP+TN+FP+FN}$$

where TP represents true positives, TN true negatives, FP false positives, and FN false negatives. These parameters were selected to understand each model’s capability to handle class imbalance and differentiate among multiple caries stages.

### 3.1. ExplainableDentalNet: Model Performance

The first one of the proposed models, ExplainableDentalNet, presented high classification performance on the test dataset, achieving an accuracy of 96.66%, with a precision of 96.70%, a recall of 96.70, and an F1-score of 96.70%. As shown in Table 2, on the Early-Stage Enamel Caries class, the model achieved 96.90% precision, 94.00 recall, and 95.40% F1-score, which highlights a strong capability to identify early lesions despite minor overlaps with the Advanced class. On the Advanced Enamel Caries class, the model obtained a precision of 93.30, a recall of 97.00, and an F1-score of 95.10, which shows reliable sensitivity in detecting severe enamel degradation. On the No Enamel Caries class, it achieved the best performance, with a precision of 1.00, a recall of 99.00, and an F1-score of 99.50, which reflects superior discrimination between samples with and without enamel caries.

### 3.2. Interpretable ResNet50-SE: Model Performance

The Interpretable ResNet50-SE model demonstrated exceptional performance, slightly surpassing that of ExplainableDentalNet in overall accuracy and precision. On the test set, this model achieved an accuracy of 98.30%, a precision of 98.40%, a recall of 98.30, and an F1-score of 98.30, as shown in Table 2. Specifically, the model achieved a precision of 100.00% on the Early-Stage Enamel Caries class, with an F1-score of 98.47%, while on the Advanced Enamel Caries class, it achieved 95.23% precision and 100% recall, with an F1-score of 98.98%. The model also performed remarkably well on the No Enamel Caries class, with 100% precision, 98.00% recall, and an F1-score of 98.98%. Although the overall accuracy was high, slight variations in F1-scores across classes indicate minor sensitivity differences among lesion categories.

**Figure 4:**
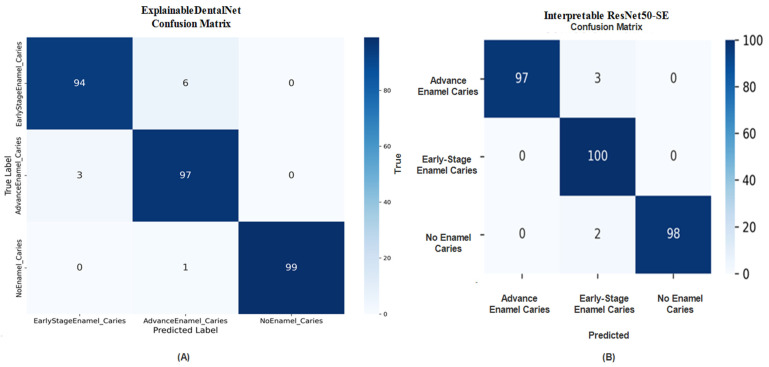
Confusion matrix (**A**) of ExplainableDentalNet presenting class-wise distribution of predictions for Early-Stage, Advanced, and No Enamel Caries. Confusion matrix (**B**) of Interpretable ResNet50-SE demonstrates high accuracy with minimal misclassifications.

The confusion matrix for ExplainableDentalNet, shown in Figure 4A, reveals strong class separation, with minimal misclassifications between Early-Stage and Advanced Enamel Caries, along with superior recognition of healthy teeth. The few misclassified samples may correspond to transitional cases where visual features are morphologically similar. The confusion matrix for Interpretable ResNet50-SE, shown in Figure 4B, supports these findings, demonstrating that most samples were correctly classified, with minor misclassifications between Advanced and No Enamel Caries. This indicates that the model is reliable, although certain visual overlaps in texture and intensity between advanced-stage and healthy enamel regions may have contributed to minor recognition errors.

**Figure 5:**
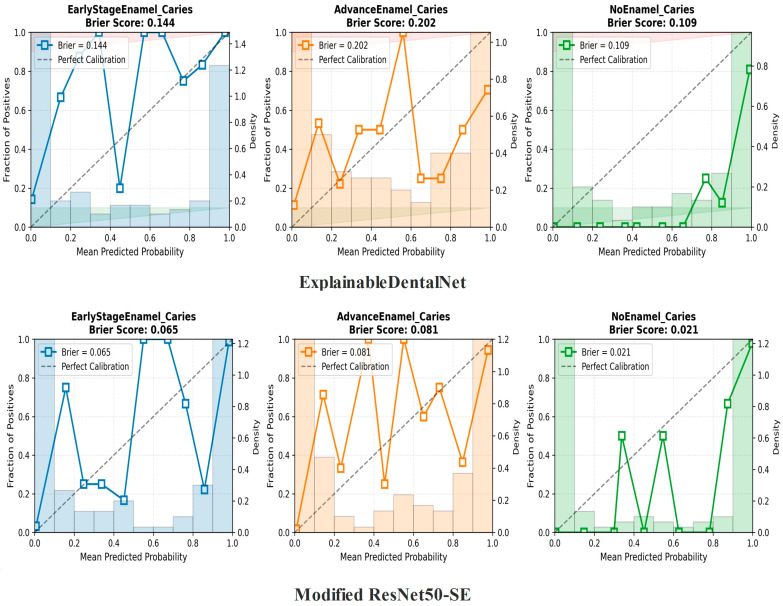
Calibration curves for the ExplainableDentalNet (**top row**) and Interpretable ResNet50-SE (**bottom row**) models across the three diagnostic classes: Early-Stage Enamel Caries, Advanced Enamel Caries, and No Enamel Caries.

The calibration plots for both models, shown in Figure 5, illustrate the relationship between the mean predicted probability (*x*-axis) and the observed proportion of positive cases (*y*-axis) for the corresponding class. A perfectly calibrated classifier would follow the reference diagonal line. The Brier score, where lower values indicate better calibration, was used to quantify the overall calibration error.

The Modified ResNet50-SE model demonstrated superior probability calibration, achieving consistently lower Brier scores across all classes (Early-Stage: 0.065; Advanced: 0.081; No Caries: 0.021) than the ExplainableDentalNet model (Early-Stage: 0.144; Advanced Enamel Caries: 0.202; No Enamel Caries: 0.109). These results indicate that the predicted probabilities generated by Interpretable ResNet50-SE more accurately reflect the true likelihood of each clinical condition.

**Figure 6:**
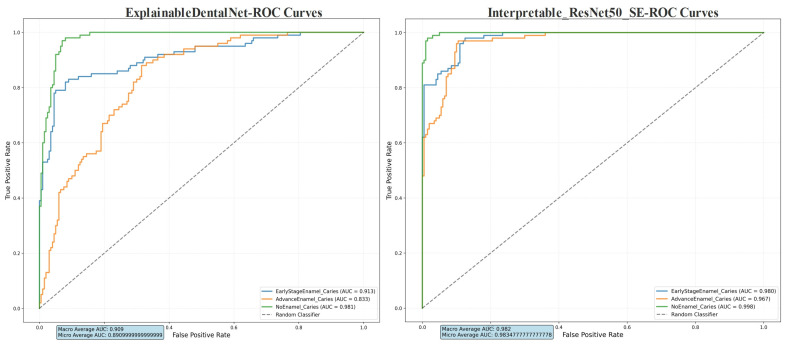
Receiver Operating Characteristic (ROC) curves for ExplainableDentalNet and Interpretable ResNet50-SE. The curves show the trade-off between sensitivity (true-positive rate) and specificity in classifying Early-Stage Enamel Caries, Advanced Enamel Caries, and No Enamel Caries Area Under the Curve (AUC) values for each class and the macro-average AUC are annotated. The dashed lines denote the performance of a random classifier (AUC = 0.5).

The Receiver Operating Characteristic (ROC) curves, presented in Figure 6, demonstrate the diagnostic discrimination capability of both models across the three classes. The Interpretable ResNet50-SE model shows superior performance, with higher Area Under the Curve (AUC) values for each class, indicating better overall ability to distinguish between enamel caries stages and sound enamel.

### 3.3. Statistical Test Evaluation

A comprehensive statistical evaluation was performed to assess the reliability and stability of both models, ExplainableDentalNet and Interpretable ResNet50-SE, on the independent test dataset containing 300 images (100 per class). Model performance was examined using McNemar’s test to detect prediction bias and non-parametric bootstrap or cross-validation analysis to estimate stability and 95.00% confidence intervals. As the evaluation was conducted on an independent dataset, paired *t*-tests were not applicable for this comparison. ExplainableDentalNet achieved an overall accuracy of 96.66% (290/300 correct), showing no significant prediction bias across classes (McNemar’s test *p* > 0.05). The bootstrap analysis indicated a mean accuracy of 0.9659 ± 0.0107 with a 95.00% CI [0.9433, 0.9833], reflecting high model stability. The Matthews Correlation Coefficient (MCC) of 0.950 further confirmed strong agreement between predicted and true enamel caries labels. The Interpretable ResNet50-SE model reached a higher overall accuracy of 98.30% (295/300 correct), also demonstrating no prediction bias (McNemar’s test *p* > 0.05). The internal cross-validation on features extracted from the test set produced a mean accuracy of 0.9900 ± 0.0082, with 95% CI [0.974, 0.999], indicating stable performance on this dataset. It is crucial to emphasize that these internal validation methods assess stability on the available data but do not guarantee generalization to new clinical settings or populations. Finally, this model’s MCC value of 0.975 indicates classification reliability. A summary of the statistical outcomes is provided in Supplementary Table S1.

### 3.4. Ablation Study

An ablation study was conducted to quantitatively assess the impact of key architectural components on Interpretable ResNet50-SE model performance and interpretability. Initially, we evaluated a baseline ResNet50 model without the Squeeze-and-Excitation (SE) attention blocks, and the results confirmed a significant decline in performance, particularly for the challenging Early-Stage Caries class. Specifically, the baseline model achieved an overall accuracy of 94.70%, with precision, recall, and F1-score for this class dropping to 95.80%, 90.00%, and 92.80%, respectively. In contrast, the full Interpretable ResNet50-SE model, with integrated SE blocks, boosted the overall accuracy to 98.30% and improved the Early-Stage Caries F1-score to 98.47%. This substantial improvement underscores the critical role of the SE mechanism in enhancing channel-wise feature recalibration, thereby focusing the model’s attention on diagnostically relevant lesion regions. Subsequently, we investigated the effect of the explainability component by removing the layer used for Grad-CAM visualization. As expected, this modification had a negligible impact on the classification metrics, with an accuracy of 98.27% and an F1-score of 98.45% on the Early-Stage Caries class, confirming that the explainability module operates post hoc and does not influence the underlying classification logic. However, its removal suppressed the model’s ability to provide visual justification for its predictions, which is a cornerstone for building clinical trust and transparency. Therefore, the explainable framework was retained as an essential component for clinical validation.

## 4. Discussion

This study demonstrates the superior diagnostic ability of the proposed ExplainableDentalNet and Interpretable ResNet50-SE models compared with recently reported methods for enamel caries detection, as shown in Table 3. The proposed architectures achieved high overall accuracy and strong class-wise consistency, confirming the benefit of combining deep convolutional feature learning with integrated explainable AI mechanisms.

ExplainableDentalNet, despite its lightweight design, effectively captured fine enamel texture variations, while the Interpretable ResNet50-SE model delivered enhanced precision and recall due to Squeeze-and-Excitation feature recalibration. The superior performance of our models can be attributed to their novel architectural features. The multi-scale feature extraction stage in ExplainableDentalNet enables the detection of both subtle texture changes and broader structural patterns in enamel, while the Squeeze-and-Excitation blocks in Interpretable ResNet50-SE adaptively recalibrate channel-wise feature responses, emphasizing diagnostically relevant caries signatures. The inclusion of Grad-CAM interpretability offers visual justification for each prediction, which is essential to clinical trust and integration into preventive dental care workflows. Unlike recent work that relied heavily on radiographic data or offered limited visual interpretability, the present framework employs intraoral clinical images and transparent AI visualization that aligns model outputs with clinical reasoning. The proposed models demonstrated competitive performance compared with prior studies, with the added benefit of explainability through Grad-CAM visualization. Although model generalizability remains limited by dataset size and diversity, the framework provides competitive performance and improved interpretability within the constraints of the evaluated dataset in enamel caries classification. Future studies should explore multimodal data fusion, e.g., combining conventional RGB images with QLF or near-infrared modalities, to enhance sensitivity for early white spot lesions and dental plaque detection, which are critical to early intervention.

### 4.1. Recommendations for Policymakers

This study provides preliminary evidence that explainable AI may support early detection workflows. However, the implications for community-level or population-level screening cannot be established without external validation and prospective clinical studies. Incorporating interpretable AI-assisted diagnostics into routine practice may also facilitate a shift from restorative treatment toward preventive, risk-based care models that align with global oral health equity goals.

#### Clinical Significance and Implications for Practice

From a clinical standpoint, the proposed explainable deep learning framework offers significant value for preventive and community-based dentistry, as it enables detection with high diagnostic precision, which in turn enables clinicians to intervene before irreversible tooth damage occurs. The models’ built-in explainability based on Grad-CAM visual maps that highlight decision-relevant regions enhances practitioners’ trust, fostering transparency and clinical acceptance. Due to its lightweight architecture and rapid inference, the proposed framework is feasible for real-time, chairside integration and portable screening applications in public dental clinics. This approach strengthens diagnostic confidence among practitioners and supports teledentistry, broadening access to quality oral healthcare, especially in low-resource populations. In future research, shifting from static classification to dynamic, temporal caries risk models leveraging follow-up images at 6- or 12-month intervals could enable the prediction of the progression of early enamel lesions to cavitation. Such model would align with the goal of preventive dentistry of individualized, risk-based care.

## 5. Conclusions

This study was to develop two explainable deep learning models, ExplainableDentalNet and Interpretable ResNet50-SE, for the automated classification of enamel caries based on intraoral images. Both models demonstrated high diagnostic performance within the constraints of the available dataset, with Interpretable ResNet50-SE showing slightly higher accuracy. The integration of Grad-CAM improved interpretability, as it highlights lesion-relevant regions, supporting clinical transparency. While the results are encouraging, the findings are limited to a single publicly available dataset and therefore do not yet indicate clinical readiness or generalizability. Future research should focus on external validation using multi-center datasets, the inclusion of patient-level metadata, and real-world clinical evaluation to determine applicability in diverse dental settings. The proposed framework provides an initial foundation for developing explainable AI tools for preventive dentistry, but further validation is essential before integration into clinical workflows

### 5.1. Limitations

This study has several limitations related to the dataset. First, the images have a low resolution, 224 × 224 pixels, which may limit the detection of subtle enamel changes. Second, the ground-truth labels, while publicly accepted, were not created by our team, and detailed annotation metadata, such as annotator experience and full ICDAS criteria application, are not provided in the dataset documentation. Third, significant data augmentation was applied to address class imbalance. Although augmentation parameters were constrained to clinically plausible ranges and the validation and test sets remained unaugmented, the possibility of synthetic bias cannot be fully excluded. Fourth, patient demographic information and clinical sampling methods for the original dataset are unavailable, preventing the analysis of potential selection biases. However, the minimal training–validation accuracy gap of ≤1.07% and cross-validation stability that overfitting was controlled. External validation could not be performed because no publicly available dataset that contains clinically captured intraoral images with the same enamel caries classes, i.e., Early-Stage, Advanced, and No Caries, currently exists. The internal validation methods used here allowed us to assess stability on the available data but do not guarantee generalization to new populations or imaging conditions. External validation is a necessary next step.

#### Future Work

In future research, we will focus on expanding the dataset with diverse clinical images and integrating multimodal data, such as 3D scans and radiographs, for comprehensive dental diagnosis. Additionally, fusing conventional RGB intraoral images with near-infrared or Quantitative Light-induced Fluorescence (QLF) modalities could improve sensitivity for early demineralization detection, as these modalities enhance contrast for white spot lesions. Another promising direction is the development of longitudinal AI models that use sequential intraoral images to predict caries progression, moving from static classification to dynamic risk assessment. Further optimization of model architecture and the use of federated learning can enhance reliability and privacy. The integration of the explainable framework into clinical decision-support systems will also be explored for real-time applications in dental diagnostics.

## Figures and Tables

**Table 1:** Summary of dataset distribution before and after augmentation.

Class Name	Original Images	Training Set (Original)	Val Set (Original)	Test Set (Original)	Augmentation Training Set	Total After Augmentation
Advanced Enamel Caries	800	600	100	100	4000	4200
Early-Stage Enamel Caries	800	600	100	100	4000	4200
No Enamel Caries	400	200	100	100	4000	4200
Total	2000	1400	300	300	12,000	12,600

**Table 2:** Performance of ExplainableDentalNet and Interpretable ResNet50-SE models on the test set.

Model	Class	Precision	Recall	F1-Score	Samples
ExplainableDentalNet	Early-Stage Enamel Caries	96.90	94.00	95.40	100
	Advanced Enamel Caries	93.30	97.00	95.10	100
	No Enamel Caries	100.00	99.00	99.50	100
	Overall Accuracy				96.66
	Matthews Correlation Coefficient (MCC)				95.00
Interpretable ResNet50-SE	Early-Stage Enamel Caries	100.00	97.00	98.47	100
	Advanced Enamel Caries	95.23	100.0	98.98	100
	No Enamel Caries	100.00	98.00	98.98	100
	Overall Accuracy				98.30
	Matthews Correlation Coefficient (MCC)				97.50

**Table 3:** Comparative performance of existing methods and proposed models for enamel caries detection.

Study	Dataset Type/Size	Model	Accuracy (%)	Explainability
Lee et al. [21]	Bitewing radiographs (304)	CNN + U-Net	90.4	None
ForouzeshFar et al. [25]	Bitewing images (713)	VGG19	93.9	None
Kang et al. [24]	Intraoral images (2682)	ResNet-50 ensemble	94.0	Partial
Zhang et al. [35]	Intraoral camera, 4361 images	MobileNet + U-Net	93.4	None
Tan et al. [36]	QLF intraoral 9478 images, 133 cases	EfficientNet-B3	92.0	None
ExplainableDentalNet (Proposed)	Intraoral photos (2000)	Custom CNN + Grad-CAM	96.7	Yes
Interpretable ResNet50-SE (Proposed)	Intraoral photos (2000)	ResNet50 + SE + Grad-CAM	98.3	Yes

## Data Availability

The intraoral image dataset used in this study is publicly available on Mendeley Data [30] under a Creative Commons Attribution 4.0 International (CC BY 4.0) license. The code and implementation files developed for this study are openly available on Mendeley Data [37] at https://doi.org/10.17632/phtw6rmwzd.1 (accessed on 12 November 2025).

## Supplementary Materials

The following supporting information can be downloaded at https://www.mdpi.com/article/10.3390/jcm14248959/s1, Figure S1: The architecture of the ExplainableDentalNet model illustrates the convolutional layers, multi-branch feature extraction, residual and dilated blocks, and GradCAM integration for explainability; Figure S2: The architecture of the Interpretable ResNet50-SE model, combining a ResNet50 backbone with Squeeze-and-Excitation (SE) blocks and dual pooling feature fusion, emphasized channel recalibration, and interpretability; Table S1: Summary of statistical analyses, such as McNemar’s test and stability CIs, for the ExplainableDentalNet and Interpretable ResNet50-SE models demonstrating reliability and class-wise consistency.
