# Supplementary material for: Automated Classification of Enamel Caries from Intraoral Images Using Deep Learning Models: A Diagnostic Study
Source: J Clin Med. 2025 Dec 18;14(24):8959. doi: 10.3390/jcm14248959 (PMC12734310; doi:10.3390/jcm14248959)
Supplement: Supplementary file 1 [file jcm-14-08959-s001.zip › jcm-4035346-supplementary.pdf]

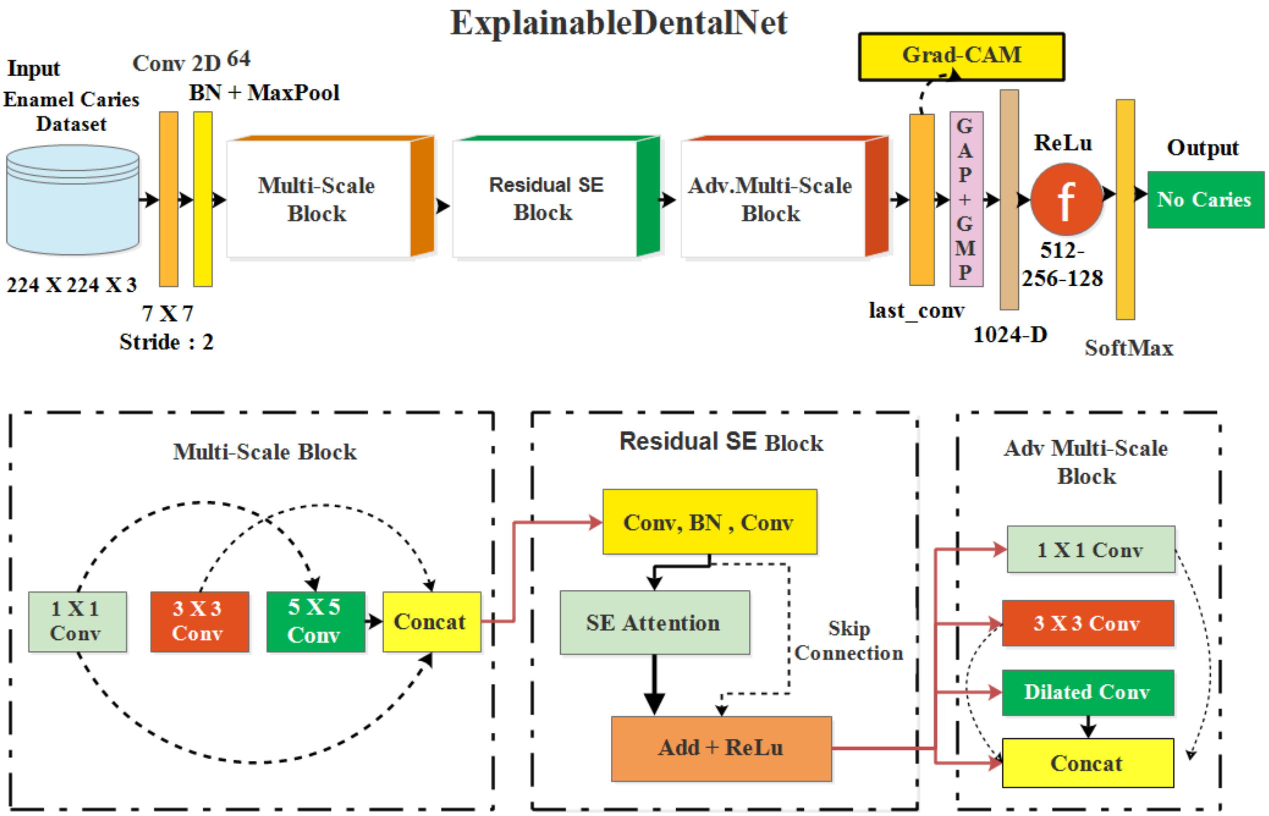

Supplementary Figure S1. The architecture of the ExplainableDentalNet model illustrates the convolutional layers, multi-branch feature extraction, residual and dilated blocks, and GradCAM integration for explainability.

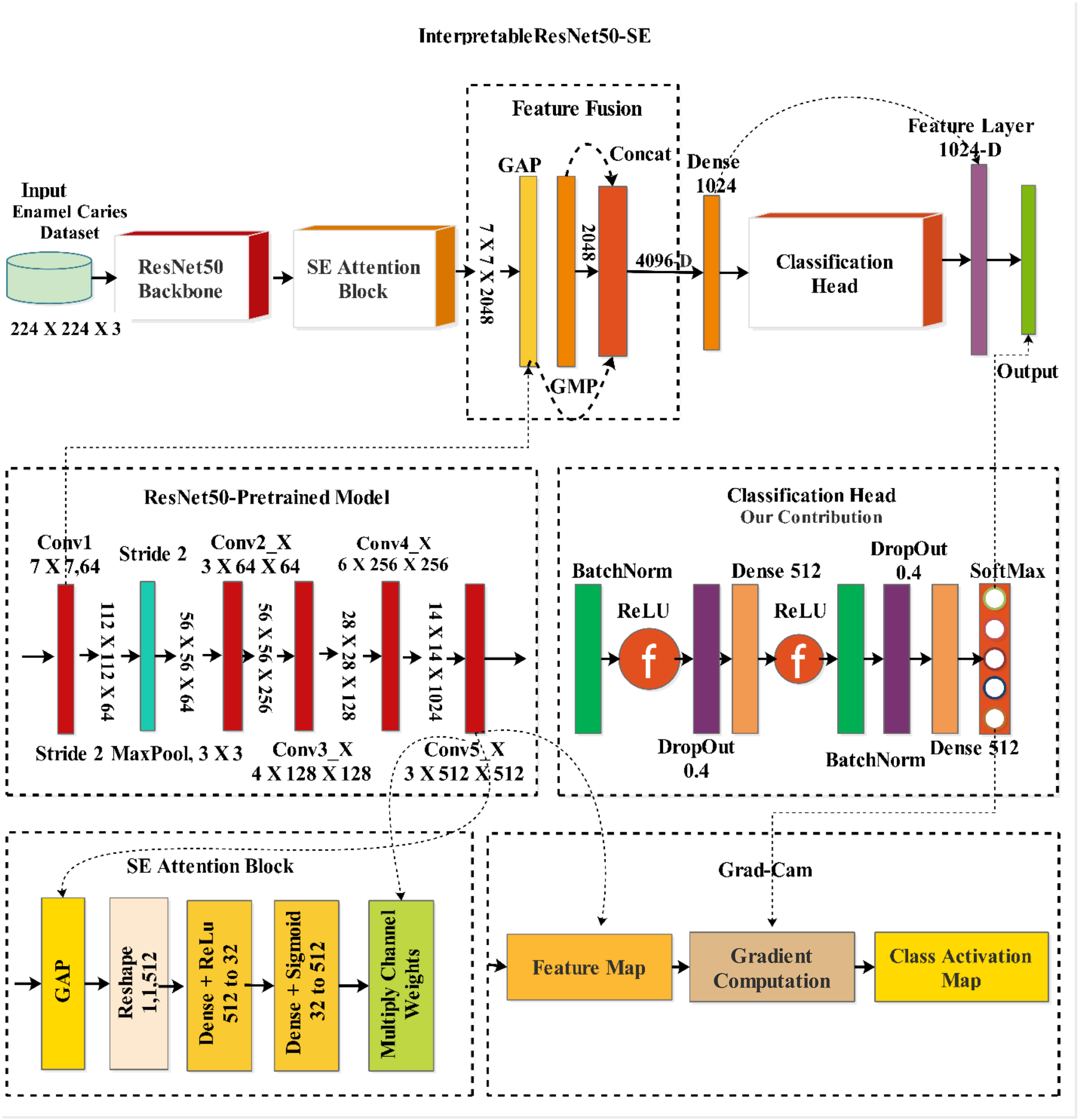

Supplementary Figure S2. The architecture of the Interpretable ResNet50-SE model, combining a ResNet50 backbone with Squeeze-and-Excitation (SE) blocks and dual pooling feature fusion, emphasized channel recalibration, and interpretability.

Supplementary Table S1. *Summary of statistical analyses, such as McNemar's test and stability CIs, for the ExplainableDentalNet and Interpretable ResNet50-SE models demonstrating reliability and class-wise consistency.*

Model	Overall Accuracy (%)	McNemar's test *p*-Value	Significant Difference	Prediction Bias	Stability (± SD)	95% CI	Stability Level

**ExplainableDental Net**	96.66	> 0.34 (NS)	None	None	0.9659 ± 0.0107	[0.9433 – 0.9833]	High
**Interpretable ResNet50-SE**	98.30	> 0.06 (NS)	None	None	0.9900 ± 0.0082	[0.974–0.999]	High
